# *Clostridium butyricum* Alleviates Enterotoxigenic *Escherichia coli* K88*-*Induced Oxidative Damage Through Regulating the p62-Keap1-Nrf2 Signaling Pathway and Remodeling the Cecal Microbial Community

**DOI:** 10.3389/fimmu.2021.771826

**Published:** 2021-11-11

**Authors:** Haihua Li, Zhiyuan Shang, Xuejiao Liu, Yingying Qiao, Kewei Wang, Jiayun Qiao

**Affiliations:** ^1^ Tianjin Key Laboratory of Agricultural Animal Breeding and Healthy Husbandry, College of Animal Science and Veterinary Medicine, Tianjin Agricultural University, Tianjin, China; ^2^ Tianjin Key Laboratory of Animal and Plant Resistance, College of Life Sciences, Tianjin Normal University, Tianjin, China; ^3^ Faculty of Biology and Technology, Sumy National Agrarian University, Sumy, Ukraine

**Keywords:** *Clostridium butyricum*, enterotoxigenic *Escherichia coli* K88, oxidative damage, p62-Keap1-Nrf2 signaling pathway, cecal microbiota, mice

## Abstract

*Clostridium butyricum* (CB) can enhance antioxidant capacity and alleviate oxidative damage, but the molecular mechanism by which this occurs remains unclear. This study used enterotoxigenic *Escherichia coli* (ETEC) K88 as a pathogenic model, and the p62-Keap1-Nrf2 signaling pathway and intestinal microbiota as the starting point to explore the mechanism through which CB alleviates oxidative damage. After pretreatment with CB for 15 d, mice were challenged with ETEC K88 for 24 h. The results suggest that CB pretreatment can dramatically reduce crypt depth (CD) and significantly increase villus height (VH) and VH/CD in the jejunum of ETEC K88-infected mice and relieve morphological lesions of the liver and jejunum. Additionally, compared with ETEC-infected group, pretreatment with 4.4×10^6^ CFU/mL CB can significantly reduce malondialdehyde (MDA) level and dramatically increase superoxide dismutase (SOD) and glutathione peroxidase (GSH-Px) levels in the serum. This pretreatment can also greatly increase the mRNA expression levels of tight junction proteins and genes related to the p62-Keap1-Nrf2 signaling pathway in the liver and jejunum in ETEC K88-infected mice. Meanwhile, 16S rDNA amplicon sequencing revealed that *Clostridium disporicum* was significantly enriched after ETEC K88 challenge relative to the control group, while *Lactobacillus* was significantly enriched after 4.4×10^6^ CFU/mL CB treatment. Furthermore, 4.4×10^6^ CFU/mL CB pretreatment increased the short-chain fatty acid (SCFA) contents in the cecum of ETEC K88-infected mice. Moreover, we found that *Lachnoclostridium*, *Roseburia*, *Lactobacillus*, *Terrisporobacter*, *Akkermansia*, and *Bacteroides* are closely related to SCFA contents and oxidative indicators. Taken together, 4.4×10^6^ CFU/mL CB pretreatment can alleviate ETEC K88-induced oxidative damage through activating the p62-Keap1-Nrf2 signaling pathway and remodeling the cecal microbiota community in mice.

## Introduction

Oxidative stress occurs when the production rate of free radicals in the body [mainly reactive oxygen species (ROS) and reactive nitrogen species (RNS)] exceeds the elimination rate of the body’s antioxidant system, resulting in an imbalance in oxidant and antioxidant effects (1). This induces lipid peroxide production, cross-linking of DNA or RNA, oxidative impairment, and protein configuration changes, which can cause dysfunction and diseases of tissues and cells. Oxidative stress can occur in response to various conditions. For example, when pathogens invade the animal body, adenosine triphosphate (ATP) is produced through the electron transport chain to provide immune response, but the body also produces a large quantity of ROS, inducing oxidative stress (2, 3). Additionally, when a pathogen is ingested, glycolysis is activated to increase the consumption of molecular oxygen, which in turn promotes the accumulation of ROS (2, 3). Enterotoxigenic *Escherichia coli* (ETEC) K88 is a gram-negative bacterium with adhesive flagella that can attach to intestinal epithelial cells (IECs), promote secretion of pro-inflammatory factors by IECs, and cause intestinal inflammation (4). After colonization, ETEC can produce enterotoxins that damage intestinal epithelial cells, causing the spread of pathogens to other organs, such as the spleen and liver (5, 6). Yacoub et al. (7) found that ETEC infections can increase liver enzyme levels, and induce reversible and irreversible liver damage. An important feature of ETEC infection is breakdown of the oxidation/antioxidant balance accompanied by the appearance of ROS (8). ROS, which are the main cause of impairment of normal cells and tissues, have been shown to be an inevitable result of the development of various diseases. Studies have shown that ETEC can promote the metabolism of methionine and cysteine, and produce toxic homocysteine, which induces oxidative stress in the body (9). Therefore, ETEC can cause oxidative stress in the animal body. Accordingly, relieving oxidative stress has become a potential method of reducing the symptoms of ETEC infection.

Recent studies have shown that the Kelch-like ECH-associated protein-1 (Keap1)-NF-E2-related factor 2 (Nrf2)/antioxidant response element (ARE) signaling pathway is a redox sensitive signal system and can regulate the body in the redox state and maintain the body stability. Nrf2 can respond to oxidative stress, is an important transcription factor that regulates oxidative stress in cells, and promotes the production of proteins involved in anti-oxidative stress, thereby exerting an antioxidant effect (10). Keap1 is a regulator of oxidative stress that combines with Nrf2 in the cytoplasm to fix Nrf2 in the cytoplasm and combines with E3 ubiquitination ligase to promote the ubiquitination and degradation of Nrf2. When the body is subjected to oxidative stress, Keap1 is inactivated, inhibits the ubiquitination and degradation of Nrf2, promotes Nrf2 accumulation, activates Nrf2, enters the nucleus, binds with ARE, and induces transcription and expression of downstream antioxidant enzyme genes to activate the defense system and alleviate oxidative damage (11, 12). In addition, the crossregulation between the Keap1-Nrf2 pathway and autophagy can resist oxidative stress (12). p62 is a substrate and active molecule involved in autophagy that can competitively bind to Keap1 with Nrf2, promote Nrf2 nuclear transfer, activate the Nrf2/ARE signaling pathway, and then exert antioxidant effects and maintain the body’s barrier function (11–13). And it has been pointed out that intestinal microbiota and their metabolites are part of the intestinal ecosystem, which together maintain the intestinal health of humans and animals. The host provides colonization sites for intestinal microbiota, and intestinal microbiota help the intestine resist invasion by harmful substances, such as pathogens (14). Intestinal microbiota can interact with the host to maintain the integrity of the tissue morphology and barrier function and promote intestinal health (14). Therefore, effective regulation of p62-Keap1-Nrf2 signaling pathway and restoration or maintenance of intestinal microbiota may be an effective strategy to alleviate ETEC K88-induced oxidative damage.

*Clostridium butyricum* (CB)is a strict anaerobe that can resist acid and tolerate high temperatures. This organism can colonize the cecum and colon, and can produce beneficial substances including a variety of digestive enzymes, vitamin B, and short-chain fatty acids (SCFAs) in its metabolic process (15, 16). Zhao et al. (17) found that CB can effectively reduce the inflammatory response and epithelial barrier damage in *Salmonella*-infected chickens through *in vivo* and *in vitro* studies. Similarly, our previous study found that CB alleviated ETEC-induced inflammatory responses in weaned piglets, and confirmed that CB is a safe and effective feed additive (18). Additionally, studies have reported that CB can alleviate oxidative damage (19, 20), but its mechanism is still not very clear. Therefore, this study was conducted to establish a pathogenic model for mice challenged with ETEC K88, and to analyze the molecular mechanism of CB pretreatment in alleviating ETEC K88-induced oxidative damage in mice based on the p62-Keap1-Nrf2 signaling pathway and intestinal microbial community, in order to provide a basis for the rational use of CB in feed.

## Materials and Methods

### Experimental Materials

CB was isolated from the feces of healthy piglets and its identity confirmed based on colony morphology, Gram staining, and sequencing of its 16S rDNA. ETEC K88 was obtained from the Laboratory of College of Animal Science and Veterinary Medicine, Tianjin Agricultural University. Live ETEC K88 was inoculated into Luria–Bertani liquid medium and incubated at 37°C for 24 h. Live CB was inoculated into Reinforced Clostridial Medium, a liquid medium, and incubated at 37°C for 48 h. Bacteria liquid: glycerol (1:1) was then stored in a refrigerator at −80°C for subsequent experiments. CB and ETEC K88 were enumerated on solid medium before the experiment. Kunming mice were purchased from the China National Institute for Food and Drug Control. Male Kunming mice aged 9–10 weeks that were in good health and weighed 22–25 g were selected for the experiment.

### Experimental Designs

#### Experiment 1 Establishment of ETEC disease model

Forty-eight mice were randomly divided into three groups [control group (CONT), low-dose group (L-ETEC), and high-dose group (H-ETEC)], with sixteen replicates in each group. The mice in the CONT group were gavaged with 0.2 mL normal saline, while those in the L-ETEC group were gavaged with 0.2 mL 3.7 × 10^7^ CFU/mL ETEC K88 and those in the H-ETEC group were gavaged with 0.2 mL 3.7 × 10^8^ CFU/mL ETEC K88. After 24 h, six mice with similar body weights were randomly selected and sacrificed in each group, and their serum antioxidant indexes were detected. The optimal concentration of the ETEC disease model was then selected.

#### Experiment 2 Effects of CB on Growth Performance in Mice

Forty-eight mice were randomly divided into three groups [control group (CONT), low-dose group (L-CB), and high-dose group (H-CB)], with sixteen replicates in each group. The mice in the CONT group were gavaged with 0.2 mL normal saline, while those in the L-CB group were gavaged with 0.2 mL 4.4 × 10^5^ CFU/mL CB and those in the H-CB group were gavaged with 0.2 ml 4.4 × 10^6^ CFU/mL CB. All of the mice were gavaged once daily at 13:00 for 15 d. The weight of the mice was recorded on days 0, 7, and 14, after which the optimal concentration of CB was selected.

#### Experiment 3 Mechanism of CB alleviation of ETEC K88-induced oxidative damage in mice

Ninety-six mice were randomly divided into 6 groups, with 16 mice in each group. Mice in the CONT group were gavaged with 0.2 mL normal saline on days 1-16; mice in the H-ETEC group were gavaged with 0.2 mL normal saline on days 1-15 and with 0.2 mL 3.7 × 10^8^ CFU/mL ETEC K88 on day 16; mice in the L-CB group were gavaged with 0.2 mL 4.4 × 10^5^ CFU/mL CB on days 1-16; mice in the H-CB group were gavaged with 0.2 mL 4.4 × 10^6^ CFU/mL CB on days 1-16; mice in the L-CB+H-ETEC group were gavaged with 0.2 mL 4.4 × 10^5^ CFU/mL CB on days 1–15 and with 0.2 mL 3.7 × 10^8^ CFU/mL ETEC K88 on day 16; mice in the H-CB+H-ETEC group were gavaged with 0.2 mL 4.4 × 10^6^ CFU/mL CB on days 1–15 and 0.2 mL 3.7×10^8^ CFU/mL ETEC K88 on day 16. Six mice with similar body weight were randomly selected and sacrificed in each group at 24 h after ETEC K88 challenge. The morphological structures of the liver and jejunum were observed, and the serum oxidation indexes and mRNA expression levels of the tight junction proteins and genes related to the p62-Keap1-Nrf2 signaling pathway in the liver and jejunum were determined.

Mice in the CONT, H-ETEC, H-CB and H-CB+H-ETEC groups were selected for analysis of the microbial community structure and its changes, and the contents of short-chain fatty acids (SCFAs) were determined.

The mouse cages were disinfected before the test, and mice were allowed to adapt to the environment for 3 d. During the experiment, all mice were allowed to drink and eat freely, and the temperature and humidity were controlled within ranges of 18–22°C and 50–60%, respectively; moreover, there was a 12 h light/dark cycle, and good ventilation was maintained. The maintenance feed for mice was purchased from Beijing Keao Xieli Feed Co, Ltd (Beijing, China). The composition of the feed ingredients: corn, soybean meal, fish power, flour, bran, NaCl, calcium hydrogen phosphate, stone powder, a variety of vitamins, a variety of trace elements, amino acids, etc. The guaranteed values for product composition analysis are shown in **Table S1** (**Supplementary Material**).

### Sample Collection and Processing

Blood was collected from the retrobulbar venous sinus of mice and placed in ice for 2–3 h. Next, sera were obtained by centrifugation at 7,000 rpm and 4°C for 20 min, after which samples were stored at −20°C until determination of oxidation indexes. After the blood was collected, the mice were sacrificed by cervical dislocation (note that they were fasted for 12 h before sacrifice, but they could drink freely before the blood was collected). The liver and jejunum tissues were then taken under aseptic conditions and divided into two parts. One part was fixed in 4% paraformaldehyde and stored at room temperature to be used for the observation of shape and structure of tissue, while the other was put in a Ziplock bag and then immediately placed into liquid nitrogen. Samples were then moved to a −80°C freezer and stored until subsequent determination of the mRNA expression levels of the target gene. The cecum contents were collected under aseptic conditions, placed in a 1.5 mL centrifuge tube, and stored in a refrigerator at −80°C until use for the determination of SCFAs and the extraction of intestinal genomic DNA.

### Observation of Tissue Histopathology

Mice liver and jejunum tissues were fixed in 4% paraformaldehyde for at least 48 h at room temperature. After dehydration in the different concentrations of ethanol, the tissues were transparent with xylene, embedded in paraffin wax and then sectioned. Paraffin section (thickness usually 3-5 μm) were stained with hematoxylin and eosin (H&E) for routine examination. Some main steps were described below. First, the paraffin sections were dewaxed with xylene, hydrated with different concentrations of ethanol and washed with distilled water. Second, the sections were incubated in hematoxylin and eosin solution, and rinsed with distilled water. Third, the sections were dehydrated, and ethanol was removed with xylene. Finally, the sections were sealed by neutral balsam, and observed using an ECHO Revolve Hybrid microscope.

Additionally, the ECHO app was used to measure villus height (VH) and crypt depth (CD) in the jejunum tissue. The VH was measured from the villus tip to the bottom. The CD was measured from the crypt tip to the bottom. An average of twelve villi and crypts was expressed as a mean VH and CD for each mouse.

### Detection of Serum Oxidation Indices

The content of malondialdehyde (MDA) in the serum was determined by the TBA colorimetric method, the content of superoxide dismutase (SOD) in the serum was determined by the xanthine oxidase method, and the content of glutathione peroxidase (GSH-Px) in the serum was determined by visible light colorimetry. The MDA, SOD and GSH-Px assay kits were purchased from Nanjing Jiancheng Biology Engineering Institute (Nanjing, China). Specific testing methods referred to Sun et al. (21).

### Quantitative Real-Time Polymerase Chain Reaction (qRT-PCR)

After the frozen liver and jejunum tissues of mice were homogenized in liquid nitrogen, the RNA in the tissues was extracted with TRIzol reagent (Takara Biotechnology, Dalian, China) according to the manufacturer’s instructions. Following reverse transcription of RNA into cDNA, qPCR was performed using the primers shown in **Table 1**. Among them, *p62* and *Nrf2* specific primers were designed using primer 5 and the full sequences of their genes obtained from NCBI. *p62*, *Nrf2*, *Heme oxygenase-1* (*HO-1*) (22), *GSH-Px* (23), *SOD1* (24), *SOD2* (22), *claudin 1* (25), *claudin 8* (26), *occludin* (27), and *zonula occludens-1* (*ZO-1*) (25) mRNA expression levels were measured using LightCycler^®^ 480 real-time PCR and calculated based on 2^-ΔΔCt^. Data are shown as ratios of abundance of target gene transcripts in the treated mice to those in the control group after normalization to *Glyceraldehyde-3-phosphate dehydrogenase* (*GAPDH*) (25). Kits for nucleic acid extraction, reverse transcription, and qRT-PCR were purchased from GeneCopoeia (Rockville, USA).

**Table 1:** Primer sequences information.

Gene names	Primer sequence/(5’→3’)	Product length/bp	Annealing temperature/°C	GenBank accession number
*p62*	F: CAACTGTTCAGGAGGAGACGA R: CTGGTGGCAGATGTGGGTA	179	62	XM_036156414.1
*Nrf2*	F: CAGTGCTCCTATGCGTGAA R: GCGGCTTGAATGTTTGTC	109	56	NM_010902.4
*HO-1*	F: CAAGGAGGTACACATCCAAGCC R: TACAAGGAAGCCATCACCAGCT	102	56	NM_010442.2
*GSH-Px*	F: GAAGTGCGAAGTGAATGG R: TGTCGATGGTACGAAAGC	224	56	X03920.1
*SOD1*	F: AACCAGTTGTGTTGTGAGGAC R: CCACCATGTTTCTTAGAGTGAGG	139	56	NM_011434.2
*SOD2*	F: ACGCCACCGAGGAGAAGTACC R: CGCTTGATAGCCTCCAGCAACTC	181	62	NM_013671.3
*Claudin 1*	F: GAGTCTCCGGTGCATCATTT R: CAGCTTGCTAGGGAACTTGG	143	56	NM_001379248.1
*Claudin 8*	F: GTGCTGCGTCCGTCTTGGCT R: TCGTCCCCCGTGCATCTGGT	79	62	NM_018778.3
*Occludin*	F: CCTTCTGCTTCATCGCTTCCTTA R: CGTCGGGTTCACTCCCATTAT	164	56	NM_001360538.1
*ZO-1*	F: GGGAGGGTCAAATGAAGACA R: GGCATTCCTGCTGGTTACAT	145	56	XM_036152895.1
*GAPDH*	F: GCACAGTCAAGGCCGAGAAT R: GCCTTCTCCATGGTGGTGAA	151	56/62	XM_036165840.1

F, shows forward primer; R, shows reverse primer.

### 16S rDNA Amplicon Sequencing for Cecum Microbiota

After the collected samples were thawed, the genomic DNA of the mice ceca was extracted by the cetyltrimethyl ammonium bromide (CTAB) method, then stored in the refrigerator at low temperature (−20°C) until further PCR amplification and sequencing.

The following 16S rRNA gene primers were used to amplify the 16S rRNA gene V3-V4 region of cecum microbiota: 341F: 5’-CCTAYGGGRBGCASCAG; 806R: 5’-GGACTACNNGGGTATCTAAT-3’. Following amplification, 2% agarose gel electrophoresis was used to detect the purity and concentration of the extracted genomic DNA. PCR products were then purified and recovered with a PCR extraction kit for quantitative determination (QIAGEN, Germany). Finally, the amplified PCR products were sequenced on the Illumina NovaSeq 6000 platform by Beijing Novogene Biology Information Technology Co., Ltd (Beijing, China). All raw data have been deposited in NCBI BioProject (accession number: PRJNA765776).

### Detection of Short-Chain Fatty Acids (SCFAs)

After the collected samples were thawed, about 0.5 g of the cecal content was put into 1.5 mL centrifuge tubes. Next, 1.25 mL of ultrapure water was added, after which the samples were vortexed for 3–5 min. Samples were subsequently centrifuged at 5,000 rpm for 10 min, after which 1.5 mL of the supernatant was collected and placed in a centrifuge. To the tubes, 0.2 mL of 25% metaphosphoric acid solution was added at a ratio of 5: 1. Samples were subsequently shaken well, placed in an ice-water bath for 30 min, then centrifuged for 10 min at 10,000 rpm at 4°C for 10 min. The supernatant was then collected, after which the contents of butyrate, acetate, propionate and total SCFAs in the cecum contents were determined by gas chromatography (Agilent 7890B). Gas chromatographic conditions were described below. The separation was performed on a DB-FFAP (30 m × 0.25 μm × 0.25 mm) column. The injector temperatures were 220°C, and the carrier gas (nitrogen) was injected at a rate of 1.0 mL/min. The detector temperatures were 250°C. The initial temperature was 100°C and the temperature was risen at 30°C per minute for 6 min.

### Statistical Analysis

SPSS 22.0 was used to perform one-way ANOVA of the growth performance, oxidation index, relative gene expression, and cecal short-chain fatty acids of mice in different groups, with Duncan’s and LSD method used to identify significant differences among groups. For nonparametric data, Mann-Whitney U test was performed.

Flash V1.2.7 (28) was used to splice the sample reads after the primer and barcode sequences to give sequences, which were then filtered and analyzed using QIIME V1.9.1. Next, Uparse v7.0.1001 (29) was used to cluster the sequences, with those having more than 97% similarity being considered the same operational taxonomic unit (OTU). Each OTU sequence was subsequently extracted and annotated using Mothur and the SSUrRNA database (30) of SILVA132 (31).

QIIME (version 1.9.1) was used to conduct α-diversity analysis (including ACE, Shannon, Coverage, Chao1) for OTUs with similarity levels greater than 97%. R (version 2.15.3) was then used to make species composition histograms and linear discriminant analysis (LDA) distribution histograms based on the OTUs abundance information to visually characterize the similarities and differences in bacterial community composition.

Spearman’s correlation values of species, oxidative stress indices, and SCFA contents were calculated using the psych package in R, and their significance was tested to identify mutual relationships between the oxidative stress index, SCFA contents, and microbial species richness (alpha diversity). The top 20 species based on abundance at the genus level were chosen for correlation analysis.

## Results

### Optimization of ETEC K88 and CB Doses

**Figure 1:**
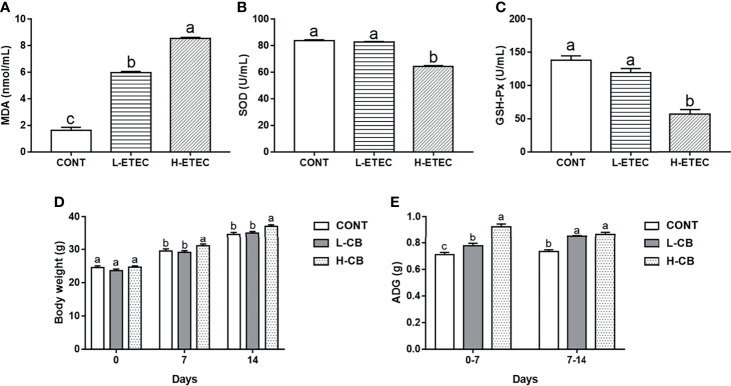
Effects of different levels of ETEC on **(A)** MDA, **(B)** SOD, and **(C)** GSH-Px in the serum of mice (n = 6 mice per group). Effects of different levels of CB on **(D)** body weight and **(E)** average daily gain of mice (n = 16 mice per group). Values shown are the means ± SEM. Different small letters indicate significant differences (*p* < 0.05).

As shown in **Figures 1A–C**, after ETEC K88 challenge, the MDA level in the serum was significantly higher than that of the CONT group (all *p* < 0.001), especially in the H-ETEC group. The SOD and GSH-Px levels in the serum in the H-ETEC group were significantly lower than those in the CONT (all *p* < 0.001) and L-ETEC groups (all *p* < 0.001). Based on the obtained results, 3.7×10^8^ CFU/mL was selected as the optimal concentration for the ETEC K88 pathogenic model.

As shown in **Figures 1D, E**, the average body weight of mice in the H-CB group was significantly higher than that of mice in the CONT (day 7 *p* = 0.038, day 14 *p* = 0.003) and L-CB groups (day 7 *p* = 0.012, day 14 *p* = 0.013) on day 7 and day 14. On days 0–7, the average daily weight gain of mice in the L-CB and H-CB groups was significantly higher than that in the CONT group (*p* = 0.017 and *p* < 0.001 respectively), and that the effects in the H-CB group were more obvious (*p* < 0.001). On days 7–14, the average daily gain of mice in the CONT group was significantly lower than that in the L-CB and H-CB groups (all *p* < 0.001). But based on the above body weight results, we are still not sure which concentration of CB has a better therapeutic effect on oxidative damage in mice. Therefore, 4.4 × 10^5^ CFU/mL and 4.4×10^6^ CFU/mL CB were used for subsequent experiments.

### CB Helps to Maintains the Integrity of Tissue Morphology Structure in Mice

**Figure 2:**
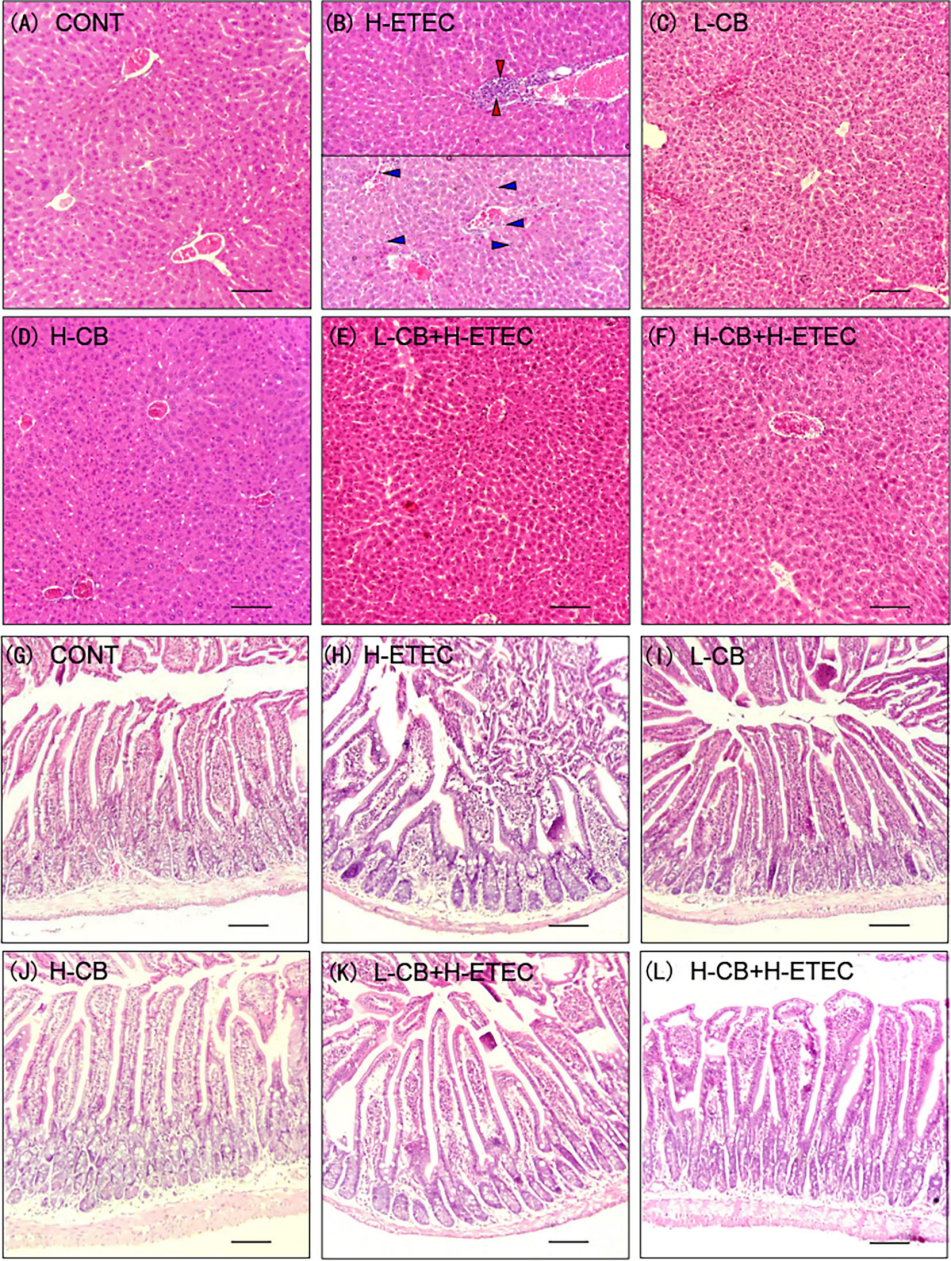
Effects of CB and/or ETEC K88 on observation of tissular morphology. **(A–F)** hematoxylin-eosin staining in the liver of mice. **(A)** CONT, **(B)** H-ETEC, **(C)** L-CB, **(D)** H-CB, **(E)** L-CB+H-ETEC, **(F)** H-CB+H-ETEC; **(G–L)** hematoxylin-eosin staining in the jejunum of mice. **(G)** CONT, **(H)** H-ETEC, **(I)** L-CB, **(J)** H-CB, **(K)** L-CB+H-ETEC, **(L)** H-CB+H-ETEC. Bar = 100 μm.

As shown in **Figures 2A–F**, the liver structure was normal with orderly arranged-hepatic in the CONT, L-CB and H-CB groups. In the liver of mice in H-ETEC group, a large number of inflammatory cells gathered around the vessels (red arrows), or inflammatory cells (blue arrows) dispersed in the vessels and hepatic sinuses, and the hepatic sinusoidal space was slightly enlarged. In the L-CB+H-ETEC and H-CB+H-ETEC groups, inflammatory cells diffused in the hepatic sinus space, and there was no large accumulation of inflammatory cells. Taken together, these results indicated that CB pretreatment can alleviate liver damage caused by ETEC K88 challenge.

As shown in **Figures 2G–L**, jejunal villi were arranged neatly in the CONT, L-CB and H-CB groups, while in the H-ETEC group, the jejunal villi became shorter, the number of inflammatory cells in villi increased, intestinal epithelial cells separated from lamina propria, and even fell off in intestinal cavity. The degree of separation and abscission of epithelial cells from lamina propria were alleviated to a certain extent in L-CB+H-ETEC and H-CB+H-ETEC groups. Taken together, these results indicated that CB pretreatment can alleviate ETEC K88-induced jejunum tissue lesions.

**Figure 3:**
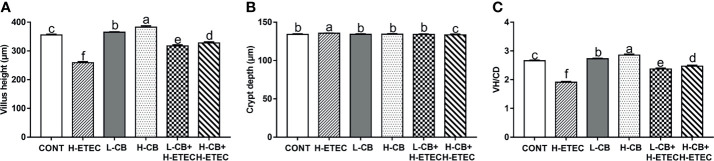
Effect of CB and/or ETEC K88 on the jejunal form of mice. **(A)** villus height, **(B)** crypt depth, **(C)** VH/CD (n = 6 mice per group, n =12 villi and crypts per mouse). Values shown are the means ± SEM. Different small letters indicate significant differences (*p* < 0.05).

As shown in **Figure 3** and **Table S2** (Supplementary material), when compared with the CONT group, ETEC K88 treatment alone could significantly increase crypt depth, reduce the jejunal villus height and VH/CD ratio (*p* = 0.031, *p* < 0.001 and *p* < 0.001, respectively). In addition, the jejunal villus height (*p* = 0.043 and *p* < 0.001, respectively) and VH/CD ratio (*p* = 0.043 and *p* < 0.001, respectively) showed significant changes after treatment with CB alone. When compared with the H-ETEC group, co-treatment with CB and ETEC K88 dramatically decreased crypt depth (*p* = 0.047 and *p* < 0.001, respectively) and significantly increased the villus height and VH/CD (all *p* < 0.001).

### CB Helps to Maintains the Soundness of Barrier Function in Mice

**Figure 4:**
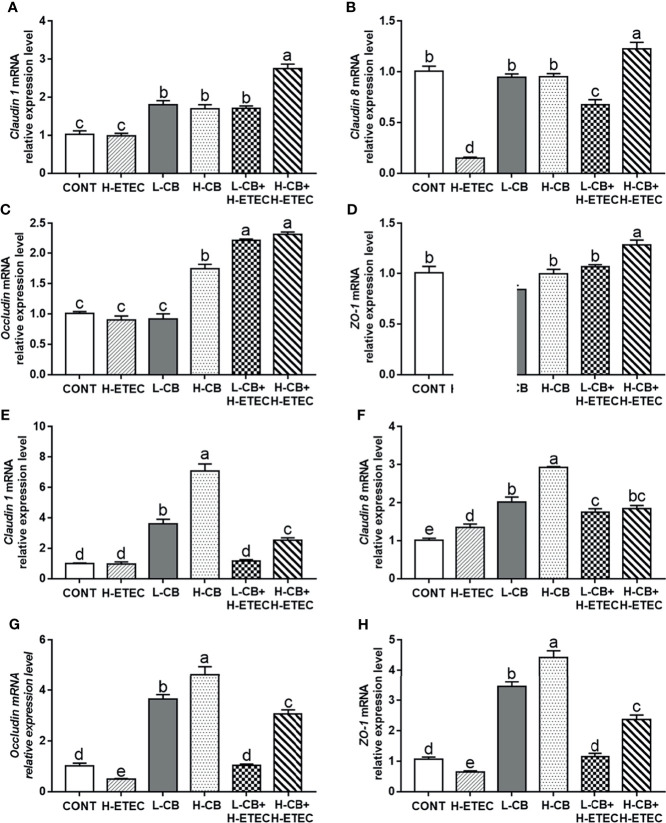
Effects of CB and/or ETEC K88 on the mRNA expression levels of tight junction proteins. **(A–D)** The mRNA expression levels of *claudin 1*, *claudin 8*, *occludin*, and *ZO-1* in the livers of mice were measured by qRT-PCR (n = 6 mice per group). **(E–H)** The expression of *claudin 1*, *claudin 8*, *occludin*, and *ZO-1* in the jejunum of mice was measured by qRT-PCR (n = 6 mice per group). Data are shown as the ratios of abundance of target gene transcripts in treated mice to those in control mice after normalization to *GAPDH*. Values shown are the means ± SEM. Different small letters indicate significant differences (*p* < 0.05).

As shown in **Figures 4A–D**, following treatment with CB alone, the mRNA expression level of *claudin 1* in the liver increased significantly (all *p* < 0.001). Additionally, the mRNA expression levels of *occludin* in the liver changed significantly after treatment with 4.4 × 10^6^ CFU/mL CB (*p* < 0.001). Following challenge with 3.7 × 10^8^ CFU/mL ETEC K88, the mRNA expression levels of *claudin 1* and *occludin* in the liver did not change significantly (*p* = 0.815 and *p* = 0.210, respectively), while the mRNA expression levels of *claudin 8* and *ZO-1* in the liver were significantly reduced (all *p* < 0.001). CB pretreatment reversed the downregulation of *claudin 8* and *ZO-1* mRNA expression induced by ETEC K88 in a dose-dependent manner, further promoted the expression of *claudin 1* and *occludin* at the transcriptional level in the liver. As shown in **Figures 4E–H**, when compared with the CONT group, the mRNA expression levels of *claudin 1* in the jejunum did not change significantly (*p* = 0.939), the mRNA expression level of *claudin 8* in the jejunum was significantly increased (*p* = 0.008), and the mRNA expression level of *ZO-1* and *occludin* in the jejunum was significantly decreased (*p* = 0.045 and *p* = 0.036, respectively) in the H-ETEC group. Additionally, in the L-CB and H-CB groups, the mRNA expression level of *claudin 1*, *claudin 8*, *occludin*, and *ZO-1* in the jejunum increased significantly (all *p* < 0.001). When compared with the H-ETEC group, the mRNA expression levels of *claudin 8* (*p* = 0.002 and *p* < 0.001, respectively), *occludin* (*p* = 0.033 and *p* < 0.001, respectively), and *ZO-1* (*p* = 0.015 and *p* < 0.001, respectively) in the jejunum were significantly increased in the L-CB+H-ETEC and H-CB+H-ETEC groups. Additionally, the mRNA expression level of *claudin 1* in the jejunum in the H-CB+H-ETEC group was significantly higher than that in H-ETEC group (*p* < 0.001). Taken together, these results indicated that 4.4 × 10^6^ CFU/mL CB pretreatment can reverse the downregulation expression of tight junction proteins (*claudin 1*, *claudin 8*, *occludin*, and *ZO-1*) at the transcriptional level in the liver and jejunum.

### CB Helps to Protect Mice Against ETEC K88-Induced Oxidative Damage Through Regulation of the p62-Keap1-Nrf2 Signaling Pathway

**Figure 5:**
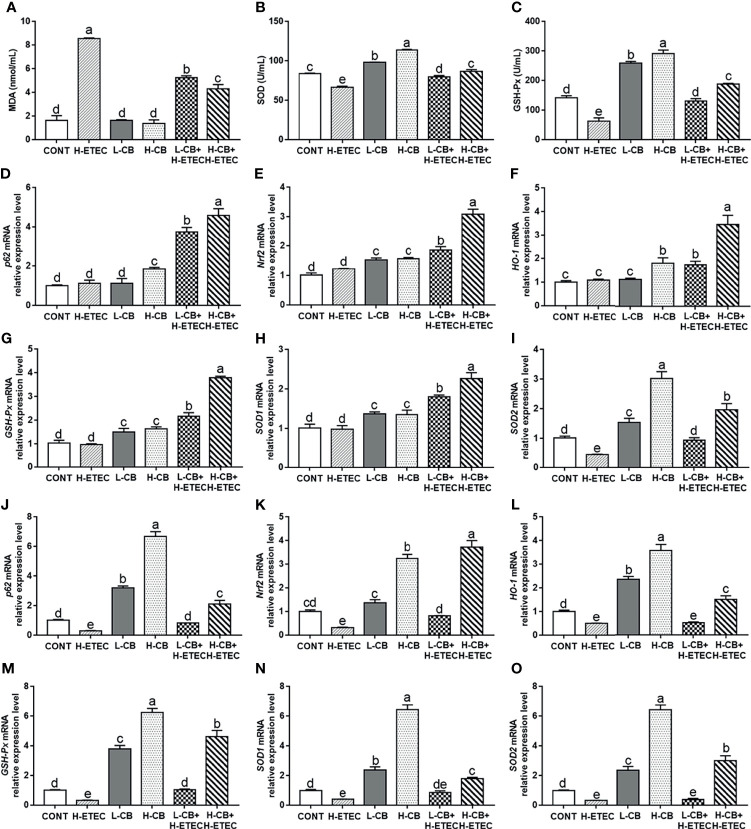
Effects of CB and/or ETEC K88 on the levels of MDA, SOD, GSH-Px, *p62*, *Nrf2*, *HO-1*, *GSH-Px*, *SOD1*, and *SOD2* in mice. **(A–C)** The levels of MDA, SOD, and GSH-Px in the serum of mice were measured (n = 6 mice per group). **(D–I)** The mRNA expression levels of *p62*, *Nrf2*, *HO-1*, *GSH-Px*, *SOD1*, and *SOD2* in the livers of mice were measured by qRT-PCR (n = 6 mice per group). **(J–O)** The mRNA expression levels of *p62*, *Nrf2*, *HO-1*, *GSH-Px*, *SOD1*, and *SOD2* in the livers of mice were measured by qRT-PCR (n = 6 mice per group). Data are shown as ratios of abundance of target gene transcripts in the treated mice to those in the control mice after normalization to *GAPDH*. Values shown are the means ± SEM. Different small letters indicate significant differences (*p* < 0.05).

As shown in **Figures 5A–C**, after treatment with CB alone, the MDA level in the serum did not differ significantly from that of the CONT group (*p* = 0.960 and *p* = 0.468, respectively), while the SOD and GSH-Px levels had increased significantly (all *p* < 0.001). Additionally, the MDA level in the H-ETEC group was significantly increased relative to the CONT group (*p* < 0.001), while the SOD and GSH-Px levels were significantly decreased (all *p* < 0.001). However, CB pretreatment significantly reduced MDA levels and increased SOD and GSH-Px levels in the serum in a dose-dependent manner in ETEC K88-infected mice, and 4.4 × 10^6^ CFU/mL CB significantly interfered with ETEC K88-induced oxidative damage in mice.

As shown in **Figures 5D–I**, the mRNA expression level of *SOD2* in the liver was significantly reduced in the H-ETEC group compared to the CONT group (*p* = 0.012). In the L-CB and H-CB group, the mRNA expression levels of *Nrf2* (*p* = 0.001 and *p* < 0.001, respectively), *GSH-Px* (*p* = 0.005 and *p* = 0.001, respectively), *SOD1* (*p* = 0.014 and *p* = 0.018, respectively), and *SOD2* (*p* = 0.018 and *p* < 0.001, respectively) in the liver were significantly increased compared to the CONT group. In addition, compared to the CONT group, the mRNA expression levels of *p62* and *HO-1* in the liver were significantly increased in the H-CB group (*p* = 0.008 and *p* = 0.007, respectively). When compared with the H-ETEC group, the mRNA expression levels of *p62* (all *p* < 0.001), *Nrf2* (all *p* < 0.001), *HO-1* (*p* = 0.024 and *p* < 0.001, respectively), *GSH-Px* (all *p* < 0.001), *SOD1* (all *p* < 0.001), and *SOD2* (*p* = 0.033 and *p* < 0.001, respectively) in the liver were significantly increased in the L-CB+H-ETEC and H-CB+H-ETEC groups in a dose-dependent fashion. As shown in **Figures 5J–O**, the mRNA expression levels of *p62*, *Nrf2*, *HO-1*, *GSH-Px*, *SOD1*, and *SOD2* in the jejunum were significantly decreased in the H-ETEC group when compared with the CONT group (*p* = 0.009, *p* = 0.002, *p* = 0.012, *p* = 0.043, *p* = 0.018 and *p* = 0.035, respectively). When compared with the H-ETEC group, co-treatment with 4.4 × 10^5^ CFU/mL CB and ETEC K88 significantly increased the *p62*, *Nrf2* and *GSH-Px* mRNA expression levels in the jejunum (*p* = 0.048, *p* = 0.020, and *p* = 0.035, respectively). Additionally, although *HO-1, SOD1*, and *SOD2* mRNA expression levels in the jejunum did not change significantly (*p* = 0.912, *p* = 0.068, and *p* = 0.867, respectively), they did show an upward trend. Furthermore, co-treatment with 4.4 × 10^6^ CFU/mL CB and ETEC K88 significantly increased the *p62*, *Nrf2*, *HO-1*, *GSH-Px*, *SOD1*, and *SOD2* mRNA expression levels in the jejunum when compared with the H-ETEC group (all *p* < 0.001). Taken together, these results indicate that CB pretreatment can increase the mRNA expression of *p62*, *Nrf2*, *HO-1*, *GSH-Px*, *SOD1*, and *SOD2* in the liver and jejunum of ETEC K88-infected mice in a dose-dependent manner.

### CB Helps to Protect Mice Against ETEC K88-Induced Oxidative Damage by Remodeling the Cecum Microbial Community Structure

PCR amplification and sequencing were performed on the 16S rDNA V3-V4 region of the genomic DNA of the cecal contents. After obtaining the raw tags of 12 samples and performing splicing, quality control, and filtering to remove the chimera, a total of 47,918 ± 2,940 effective tags, 19,785,811 ± 1,224,633 bp bases, and a 413 bp AvgLen (average length of effective tags) were obtained. Based on the minimum number of sample sequences and clustering the OTUs at 97% similarity, a total of 1296 OTUs were obtained, including 1 kingdom, 13 phyla, 21 classes, 43 orders, 72 families, 158 genera, and 141 species.

**Figure 6:**
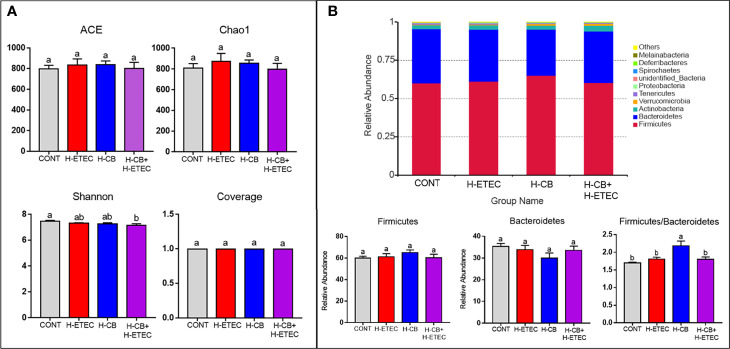
Effects of CB and/or ETEC K88 on the cecal microbial community structure. **(A)** Alpha diversity of cecal microbiota in mice (n = 3 mice per group). **(B)** Relative abundance of cecal microbiota of mice in different groups at the phylum level (n = 3 mice per group). Values shown are the means ± SEM. Different small letters indicate significant differences (*p* < 0.05).

**Figure 7:**
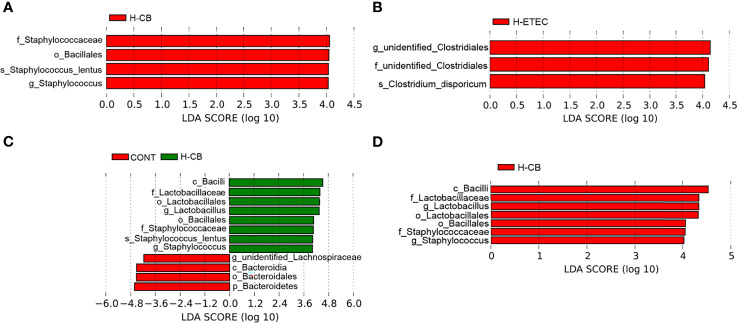
Identification of specific microbial communities under CB and/or ETEC K88. Different bacteria communities from the phylum to species level were evaluated by linear discriminant analysis coupled with effect size (LEfSe). **(A)** Among four different groups, **(B)** between CONT and H-ETEC groups, **(C)** between CONT and H-CB groups, and **(D)** among CONT, H-CB and H-CB+H-ETEC groups (n = 3 mice per group).

As shown in **Figure 6A**, the ACE and Chao1 indexes did not differ significantly among the four groups (*p* = 0.887), indicating that there was no significant difference in the abundance of cecal bacteria among treatment groups. Additionally, the coverage was around 0.99, indicating that the sequencing results fully reflected the actual situation of the microbiota. The Shannon’s index of the H-ETEC, H-CB, and H-CB+H-ETEC groups was lower than that of the CONT group, and there was a significant difference between the H-CB+H-ETEC group and CONT group (*p* = 0.021), indicating that the diversity of the bacterial community tended to decrease in ceca treated with ETEC K88 and CB. At the phylum level, the top 10 species in terms of relative abundance were selected. The mean value of abundance was then used to calculate the proportion of each bacterial group in the samples of the four groups, and relative abundance histograms of the species were made (**Figure 6B**). The results showed that Firmicutes and Bacteroidetes were the dominant phyla in all groups, and that there were no significant differences in relative abundance (*p* = 0.648 and *p* = 0.646, respectively). However, the Firmicutes/Bacteroidetes ratio in the H-CB group was significantly higher than that in the other three groups (*p* = 0.003, *p* = 0.011 and *p* = 0.010, respectively). To determine the specific cecal microbiota in the different treatment groups, the LDA score was set at 4 at the phylum-to-species classification level, LEfSe multi-level species difference discriminant analysis was performed, and an LDA distribution histogram was made (**Figures 7A–D**). Mice in the H-ETEC group showed lower abundance of Bacillales, Staphylococcaceae, *Staphylococcus*, and *Staphylococcus_lentus* than in the H-CB group (**Figure 7A**), whereas unidentified_Clostridiales, *unidentified_Clostridiales*, and *Clostridium_disporicum* in the H-ETEC group exhibited higher abundance than in the CONT group (**Figure 7B**). Additionally, mice treated with 4.4 × 10^6^ CFU/mL CB showed higher levels of Bacilli, Lactobacillales, Lactobacillaceae, *Lactobacillus*, Bacillales, Staphylococcaceae, and *Staphylococcus* than in the CONT and H-CB+H-ETEC groups, whereas *unidentified_Lachnospiraceae*, Bacteroidetes, Bacteroidia, and Bacteroidales in the H-CB group showed lower abundance than the CONT group (**Figures 7C, D**).

**Figure 8:**
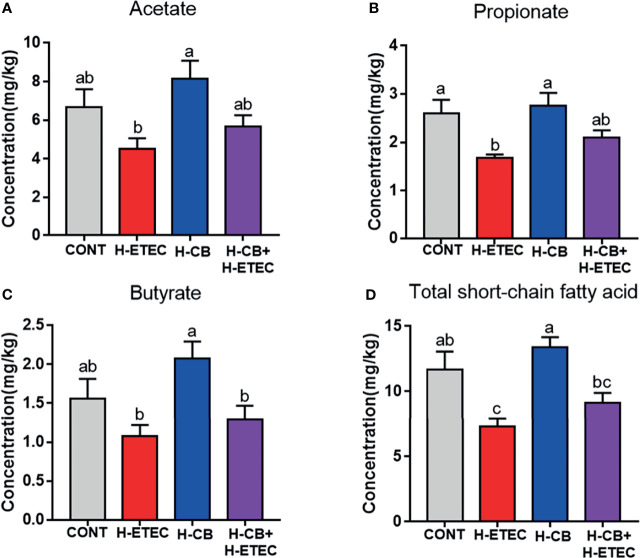
Effects of CB and/or ETEC K88 on **(A)** acetate, **(B)** propionate, **(C)** butyrate and **(D)** total short-chain fatty acids in the cecum of mice (n = 3 mice per group). Values shown are the means ± SEM. Different small letters indicate significant differences (*p* < 0.05).

As shown in **Figure 8**, the concentrations of propionate (*p* = 0.014) and total SCFAs (*p* = 0.010) in the H-ETEC group were significantly lower than those in the CONT group. When compared with the CONT group, there were no significant differences in the concentrations of acetate (*p* = 0.211), propionate (*p* = 0.602), butyrate (*p* = 0.107), and total SCFAs (*p* = 0.238) in the H-CB group. The concentrations of acetate (*p* = 0.369 and *p* = 0.328, respectively), propionate (*p* = 0.124 and *P* = 0.192, respectively), butyrate (*p* = 0.374 and *P* = 0.478, respectively), and total SCFAs (*p* = 0.082 and *p* = 0.209, respectively) in the H-CB+H-ETEC group were not significantly different compared with those in the CONT and H-ETEC groups, but the concentrations tended to increase compared with the H-ETEC group. These findings indicated that CB pretreatment can increase the concentrations of SCFAs in the cecum of ETEC K88-infected mice to a certain extent.

**Figure 9:**
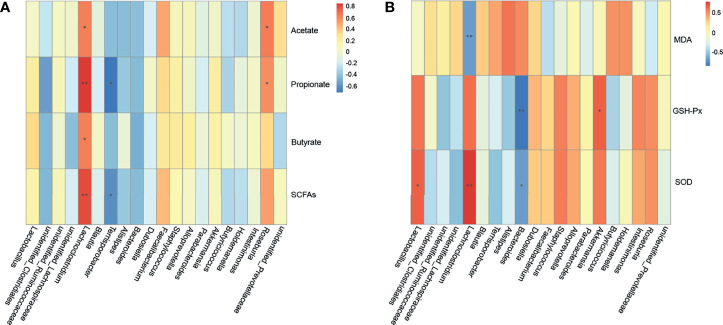
Correlation among cecal microbiota, cecal SCFAs and oxidative stress indexes. **(A)** Spearman’s correlation analyses between the cecal SCFAs and microbiota at the genus level (n = 3 mice per group). **(B)** Spearman’s correlation analyses between the cecal microbiota and oxidative stress indexes at the genus level (n = 3 mice per group). ^*^ represents the specific genus whose abundance were significantly correlated with cecal SCFAs or oxidative stress indexes. ^*^ 0.01 < *p* ≤ 0.05, ^**^ 0.001 < *p* ≤ 0.01.

Spearman’s correlation was used to analyze the oxidation indicators (MDA, SOD, GSH-Px), SCFAs, and microbiota to evaluate the correlation between ETEC K88-induced oxidative stress and changes in the intestinal microbiota and their metabolites (**Figures 9A, B**). As shown in **Figure 9A**, *Roseburia* was significantly positively correlated with acetate and propionate (*p* = 0.027 and *p* = 0.048, respectively), while *Lachnoclostridium* was significantly positively correlated with SCFA contents (*p* = 0.032, *p* < 0.001, *p* = 0.032 and *p* = 0.002, respectively). *Terrisporobacter* was found to have a significant negative correlation with propionate and total short-chain fatty acids (*p* = 0.011 and *p* = 0.042, respectively). As shown in **Figure 9B**, *Lachnoclostridium* had a significant negative correlation with MDA (*p* = 0.010) and a significant positive correlation with SOD (*p* = 0.006). In addition, *Bacteroides* was significantly negatively correlated with GSH-Px and SOD (*p* < 0.001 and *p* = 0.011, respectively), *Lactobacillus* was positively correlated with SOD (*p* = 0.033), and *Akkermansia* was positively correlated with GSH-Px (*p* = 0.033). It should be noted that *Lachnoclostridium*, *Lactobacillus*, and *Roseburia* all belong to Lachnospiraceae. Taken together, these results indicate that a positive effect of CB pretreatment on ETEC K88-infected mice may be achieved by increasing the abundance of *Lactobacillus* and the contents of SCFAs.

## Discussion

In the animal body, when the oxidative and antioxidant balance is disturbed by a pathogen infection, oxidative stress will occur (32). The integrity of tissue morphology and barrier function is one of the most important indicators used to evaluate the health of animals. Additionally, villus height and crypt depth are key factors that determine intestinal absorption and digestion of nutrients, and their ratio reflects intestinal function (33). When the intestinal villus height becomes shorter, the absorption area of the intestine decreases, which affects nutrient absorption (34). This study showed that ETEC K88 can reduce villus height and VH/CD ratio, increase crypt depth, which may affect digestion and absorption in mice. Oxidative stress has been reported to be important to impairment of barrier function. Specifically, it can change the structure of the occludin-ZO-1 complex by stimulating tyrosine phosphorylation, destroy tight junctions, and destroy barrier function (35). ETEC K88 was previously shown to induce impairment of barrier function of pig intestinal epithelial cells by reducing the expression of *ZO-1* and *occludin* (36), and these effects were confirmed in mice in the present study. This study also revealed that CB pretreatment can increase the villus height and VH/CD ratio in the jejunum of ETEC K88-infected mice, promote intestinal development, and maintain the integrity of intestinal villus structure. This pretreatment also improves expression of the tight junction protein in the liver and jejunum at the transcriptional level, maintains tissue barrier function, and alleviates oxidative damage caused by ETEC K88 in mice, which is consistent with the results reported by Chen et al. (37). Therefore, determining how CB pretreatment can improve ETEC K88-induced oxidative damage in mice was the focus of this study.

MDA, SOD, and GSH are all important markers reflecting the degree of oxidative stress. Among these, SOD and GSH are members of the body’s antioxidant system that can effectively decompose peroxides and lipid peroxides; therefore, the levels of SOD and GSH can reflect the body’s ability to scavenge oxygen free radicals (38, 39). GSH-Px can utilizes reduced GSH to convert H_2_O_2_ to water, effectively maintaining cell stability and protecting cell structure (40). MDA is a lipid metabolite that can directly reflect the intensity and rate of lipid peroxidation in the body and indirectly reflect the extent of tissue impairment by free radicals (41). This study revealed that CB pretreatment can reverse the upregulation of MDA levels and the downregulation of SOD and GSH-Px levels in the serum by ETEC K88, and alleviate oxidative damage. The p62-Keap1-Nrf2 signaling pathway is the most important endogenous antioxidant signaling pathway in the body and can regulate the expression of antioxidant enzyme genes (42). Therefore, we determined the expression of *p62*, *Nrf2*, *HO-1*, *GSH-Px* and *SODs* genes at the transcriptional level in mouse liver and jejunum. The results suggested that after treatment with ETEC K88 alone, the mRNA expression of genes related to the p62-Keap1-Nrf2 signaling pathway were all significantly lower in jejunum, while only *SOD2* mRNA expression was significantly lower in liver, indicating that jejunum is more susceptible to ETEC K88 than liver. Moreover, CB pretreatment increased the expression of *p62*, *Nrf2*, *HO-1*, *GSH-Px*, and *SODs* genes in the liver and jejunum of ETEC K88-infected mice. The upregulation of antioxidant enzyme genes is an adaptation to oxidative stress. In addition, it has been confirmed in our previous *in vitro* study that CB could activate the Nrf2/ARE signaling pathway to alleviate ETEC K88-induced oxidative damage in porcine intestinal epithelial cells (43). Therefore, it is speculated that the role of CB in alleviating oxidative damage in ETEC K88-infected mice may be related to the activation of the p62-Keap1-Nrf2 signaling pathway, and the effect in the H-CB+H-ETEC group was more obvious than that in the L-CB+H-ETEC group.

The intestinal microbiota constitutes a natural barrier against invasion by exogenous pathogens, and plays an important role in host metabolism, immunity, and evolution (44). Therefore, we used 16S rDNA amplicon sequencing to detect changes in mouse cecum microbiota. Firmicutes and Bacteroidetes dominate animal intestines, and their abundance ratio is often used as a sign of intestinal microbial disorders (45). This study found that at the phylum level, the dominant bacteria in the ceca of mice were Firmicutes and Bacteroidetes, and their ratio was significantly higher in the H-CB group, indicating CB treatment could maintain the balance of intestinal microbiota. In addition, the abundance of *Clostridium disporicum* in ETEC K88-challenged mice was significantly higher than that in the CONT group, *Staphylococcus_lentus* and *Lactobacillus* were significantly clustered in the H-CB group in this study. *Clostridium_disporicum* is a saccharolytic species within Firmicutes that exists in the ceca of mice (46) and is related to the degradation of complex organic matter (47), but its actual effects in the intestine are unclear. However, studies have shown that *Clostridium_disporicum* was found in the intestinal microbiota of patients with gastrointestinal dysfunction (48), so it is speculated that this organism may play a negative role in the intestine. However, *Lactobacillus* are gram-positive bacteria that can promote the fermentation of carbohydrates into lactic acid. Moreover, the combination of *Lactobacillus reuteri* CCM8617 and flaxseed can increase the concentration of SCFAs in the intestines of mice, maintain the integrity of the mucosa, promote the growth of intestinal epithelial cells, have a biological antagonistic effect on the pathogenic bacteria *E.coli* O149:F4, and promote intestinal health (49). It is worth noting that *Staphylococcus lentus*, which was significantly enriched in the cecum after treatment with CB, is an animal pathogen (50). Moreover, it has been reported that CB may carry virulence genes capable of producing botulinum toxin or clostridium toxin, which are pathogenic and interfere with the balance of intestinal microbiota (51). Thus, the probiotic effects of CB and factors that trigger the expression of pathogenic factors require further investigation.

The metabolites of gut microbiota, which are the result of interactions between the host and the microbiota, play key roles in maintenance of the integrity of the mucosal structure and tissue repair and can indirectly reflect the health of the body (14). SCFAs are derived from microbial fermented carbohydrates and play key roles in infection by pathogens (52). Therefore, this study determined the levels of butyrate, acetate, propionate, and total SCFAs in the cecum contents. It was found that ETEC K88 decreased the levels of SCFAs in the cecum contents, while CB pretreatment increased the levels of SCFAs to some extent. Among SCFAs, butyrate can enhance intestinal barrier function by regulating endogenous host defense peptides and promote the body to clear ETEC O157:H7 (53, 54), acetate can impair metabolic function of ETEC K88, inhibit the accumulation of toxic homocysteine, and alleviate oxidative damage (9), and propionate can inhibit the growth and reproduction of *Salmonella typhimurium* by reducing pH (55). SCFAs can be quickly absorbed by the small intestine, after which they act as substrates to participate in energy production, maintain intestinal morphology and function, improve inflammation, and relieve oxidative stress (56, 57). Therefore, we hypothesized that CB alleviation of ETEC K88-induced oxidative damage in mice might be achieved by increasing the concentrations of SCFAs.

To better understand the relationship between intestinal microbiota and their metabolites and oxidative stress, this study used Spearman’s correlation analysis to determine if there was a close relationship between Lachnospiraceae (including *Lachnoclostridium*, *Roseburia*, and *Lactobacillus*), *Terrisporobacter*, *Akkermansia*, *Bacteroides*, and SCFAs content and oxidative stress indicators. It should be noted that Lachnospiraceae are producers of SCFAs (58). The results revealed that *Terrisporobacter* is positively correlated with oxidative stress, which is consistent with the results of previous studies (59). Moreover, *Akkermansia* is a Verrucomicrobia that can promote intestinal health (60). In this study, one important finding was that the abundance of *Lactobacillus* was significantly and positively correlated with SOD, suggesting that the CB pretreatment may have alleviated ETEC K88-induced oxidative damage by increasing the abundance of *Lactobacillus*.

**Figure 10:**
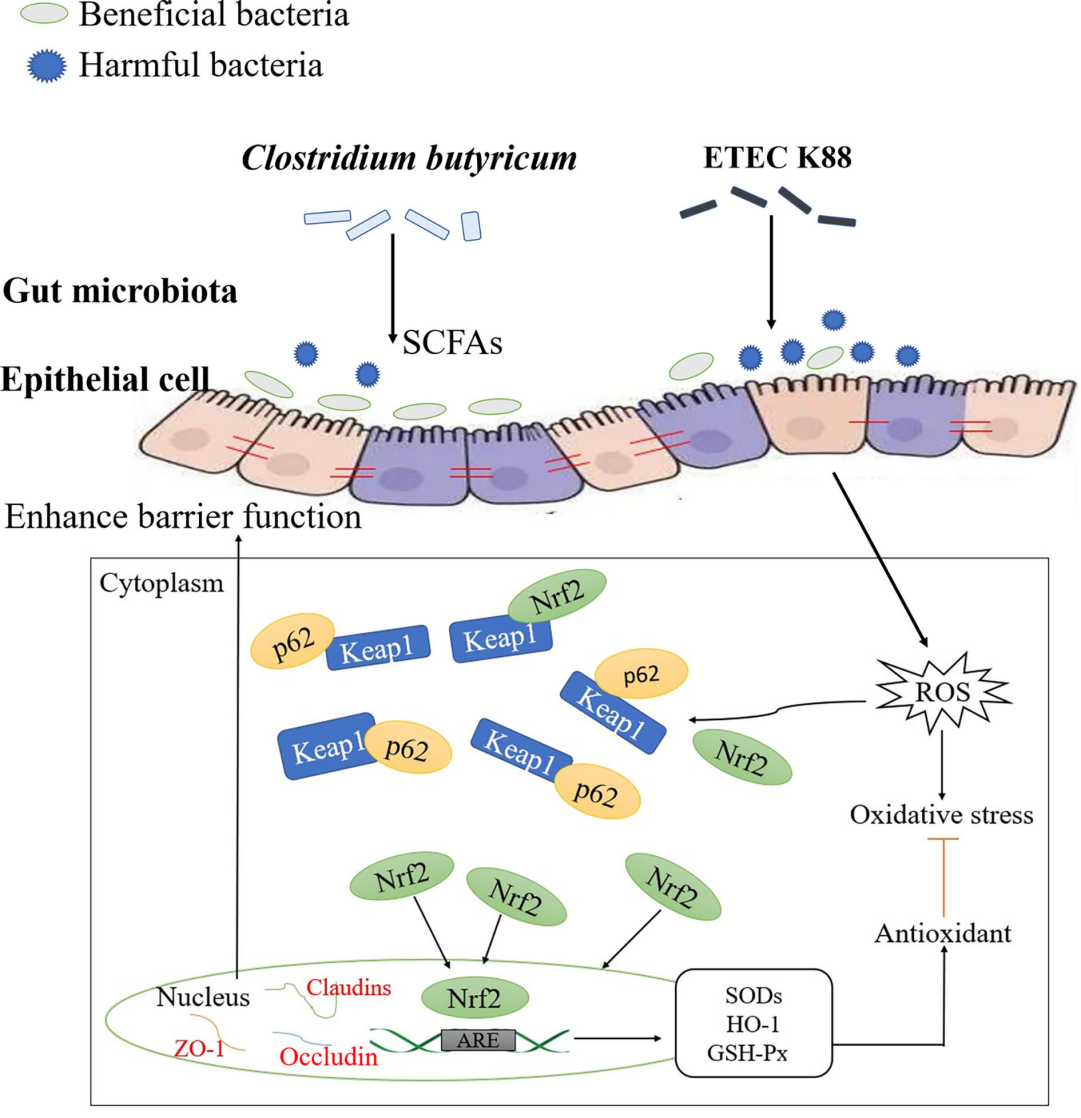
Diagram outlining the mechanism of CB on alleviating ETEC K88-induced oxidative damage.

In this study, ETEC K88 was used as the disease-causing model for *in vivo* tests. The results revealed that the alleviation of oxidative damage by CB was closely related to the p62-Keap1-Nrf2 signaling pathway and intestinal microbial community structure *via* the potential mechanism of action shown in **Figure 10**. CB can up-regulate the mRNA expression of genes related to the p62-Keap1-Nrf2 signaling pathway, promote the expression of tight junction proteins at the transcriptional level, and alleviate ETEC K88-induced oxidative damage. Additionally, it can also promote the proliferation of (*Lactobacillus*), compete with pathogenic bacteria for colonization sites, produce SCFAs, maintain body barrier function, and alleviate ETEC K88-induced oxidative damage. However, this study did not knock out the Nrf2 gene to further verify the role of the p62-Keap1-Nrf2 signaling pathway in the mitigation of oxidative damage by CB, which remains to be further investigated.

## Data Availability Statement

The original contributions presented in the study are publicly available. This data can be found here: https://www.ncbi.nlm.nih.gov/bioproject/, PRJNA765776.

## Ethics Statement

The animal study was reviewed and approved by Tianjin Agricultural University.

## Author Contributions

JQ, HL, and ZS designed the experiments. ZS, XL, YQ, and KW performed the experiments. ZS analyzed the experimental data. JQ and HL wrote this paper. All authors contributed to manuscript revision, read, and approved the submitted version.

## Funding

This project was financially supported by the Tianjin Natural Science Foundation [20JCZDJC00190] and the Tianjin “131” Innovative Talents Team [20180338].

## Conflict of Interest

The authors declare that the research was conducted in the absence of any commercial or financial relationships that could be construed as a potential conflict of interest.

## Publisher’s Note

All claims expressed in this article are solely those of the authors and do not necessarily represent those of their affiliated organizations, or those of the publisher, the editors and the reviewers. Any product that may be evaluated in this article, or claim that may be made by its manufacturer, is not guaranteed or endorsed by the publisher.

## Abbreviations

CFU, Colony forming unit; PCR, Polymerase chain reaction; MDA, Malondialdehyde; SOD, Superoxide dismutase; GSH-Px, Glutathione peroxidase; qRT-PCR, Quantitative real-time polymerase chain reaction; *ZO-1*, Zonula occludens-1; *HO-1*, Heme oxygenase-1; ROS, Reactive oxygen species; RNS, Reactive nitrogen species; Nrf2, NF-E2-related factor 2; Keap1, Kelch-like ECH-associated protein-1; ARE, Antioxidant response element; SCFAs, Short-chain fatty acids; *GAPDH*, Glyceraldehyde-3-phosphate dehydrogenase; CTAB, Cetyltrimethyl Ammonium Bromide; OTU, Operational taxonomic unit; VH, Villus height; CD, Crypt depth; ATP, Adenosine triphosphate; LDA, Linear discriminant analysis.
