# Supplementary material for: Clostridium butyricum Alleviates Enterotoxigenic Escherichia coli K88-Induced Oxidative Damage Through Regulating the p62-Keap1-Nrf2 Signaling Pathway and Remodeling the Cecal Microbial Community
Source: Front Immunol. 2021 Nov 11;12:771826. doi: 10.3389/fimmu.2021.771826 (PMC8660075; doi:10.3389/fimmu.2021.771826)
Supplement: Supplementary file 1 [file Table_1.docx]

**Table S1** Guaranteed value of product composition analysis (per kg of feed)

| Ingredients | Contents | Ingredients | Contents | Ingredients | Contents |
| --- | --- | --- | --- | --- | --- |
| Vitamin A | ≥ 7000 IU | Na | ≥ 2 g | Crude protein | ≥ 180 g |
| Vitamin D | ≥ 800 IU | K | ≥ 5 g | Crude fat | ≥ 40 g |
| Vitamin E | ≥ 6 IU | Mg | ≥ 2 g | Crude fiber | ≤ 50 g |
| Vitamin K | ≥ 3 mg | Cu | ≥ 10 mg | Lys | ≥ 8.2 g |
| Vitamin B_1_ | ≥ 8 mg | Fe | ≥ 100 mg | Met+Cys | ≥ 5.3 g |
| Vitamin B_2_ | ≥ 10 mg | Zn | ≥ 30 mg | Arg | ≥ 9.9 g |
| Vitamin B_6_ | ≥ 6 mg | Mn | ≥ 75 mg | Trp | ≥ 1.9 g |
| Vitamin B_12_ | ≥ 0.02 mg | I | ≥ 0.5 mg | His | ≥ 4.0 g |
| Biotin | ≥ 0.1 mg | Se | 0.1-0.2 mg | Phe+Tyr | ≥ 11.0 g |
| Nicotinic acid | ≥ 45 mg | Ca | 10-18 g | Thr | ≥ 6.5 g |
| Pantothenic acid | ≥ 17 mg | P | 6-12 g | Leu | ≥ 14.4 g |
| Folic acid | ≥ 4 mg | Moisture | ≤ 100 g | Ile | ≥ 7.0 g |
| Choline | ≥ 1250 mg | Crude ash | ≤ 80 g | Val | ≥ 8.4 g |

**Table S2** Effect of CB and/or ETEC K88 on the jejunal form of mice

|  | CONT | H-ETEC | L-CB | H-CB | L-CB+H-ETEC | H-CB+H-ETEC |
| --- | --- | --- | --- | --- | --- | --- |
| Villus height  (μm) | 355.58±2.92^c^ | 259.15±3.90^f^ | 365.04±1.64^b^ | 382.74±4.73^a^ | 317.56±3.90^e^ | 327.86±3.92^d^ |
| Crypt depth  (μm) | 134.09±0.81^b^ | 135.55±0.37^a^ | 134.14±0.76^b^ | 134.32±0.77^b^ | 134.02±0.79^b^ | 133.46±1.14^c^ |
| VH/CD | 2.65±0.03^c^ | 1.91±0.03^f^ | 2.73±0.02^b^ | 2.85±0.04^a^ | 2.37±0.03^e^ | 2.47±0.04^d^ |

Note: values shown are the means ± SEM (n = 6 mice per group, n = 12 per mouse). Different small letters in the same row indicate significant differences (*p* < 0.05).
